# Supplementary material for: Single cell transcriptomics identifies distinct profiles in pediatric acute respiratory distress syndrome
Source: Nat Commun. 2023 Jun 30;14:3870. doi: 10.1038/s41467-023-39593-0 (PMC10313703; doi:10.1038/s41467-023-39593-0)
Supplement: Supplementary file 1 — Supplementary Information [file 41467_2023_39593_MOESM1_ESM.pdf]

Supplementary Figure 1

a Mechanical ventilation support requirements

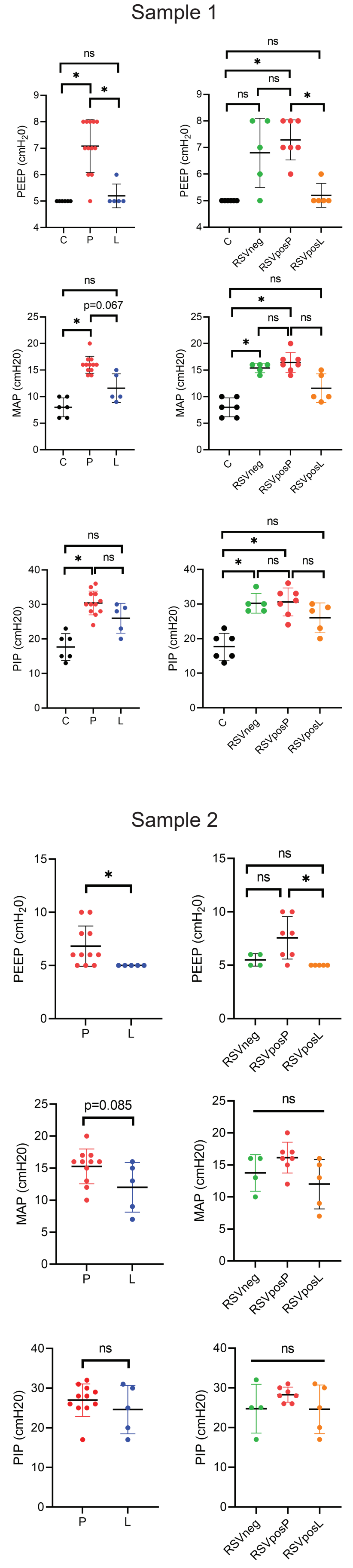

b Patient grouping for scGEX analyses

Patient	Clinical Diagnosis	Group
P001	B-*H.flu*	RSV neg
P002	V-HRV	RSV neg
P003	C-HRV,RSV; *H. flu, M. cat*	RSVposP
P004	V-ADV	RSV neg
P005	C-RSV; *H. flu, M. cat*	RSVposP
P006	C-HRV; *empiric rx*	RSV neg
P007	V-RSV	RSVposP
P008	C-RSV; *M. cat, GBS*	RSVposP
P009	C-RSV; *M. cat, S. pne*	RSVposP
P010	V-RSV	RSVposP
P011	V-IAV	RSV neg
P012	V-RSV	RSVposP
L001	V-RSV	RSVposL
L002	V-RSV	RSVposL
L003	V-RSV	RSVposL
L004	C-RSV; *H. flu, M.cat, S. pne*	RSVposL
L005	C-RSV; *S. aur, M.cat, S. pne*	RSVposL
C001	brain tumor	Con
C002	status epilepticus	Con
C003	pyloric stenosis	Con
C004	cdh	Con
C005	ingestion	Con
C007	apnea*	Con

Supplementary Figure 1: Additional Clinical Data and Patient Grouping for Single Cell Gene Expression Analyses based on clinical and ViroCap findings. a, Mechanical ventilation support requirements at the time each sample was acquired grouped by Patient enrollment cohort (left; n=6 patients for C-cohort, n=12 patients for P-cohort Sample 1, n=11 patients for P-cohort Sample 2, and n=6 patients for L-cohort) and Patient group (right; n=6 patients for C group, n=5 patients for RSVneg group Sample 1, n=4 patients for RSVneg group Sample 2, n=7 patients for RSVposP group, and n=5 patients for RSVposL group). Statistical testing was performed using a two-sided Wilcoxon Rank Sum Test (for comparisons between two groups) or a Kruskal-Wallis Test followed by Dunn's post-hoc testing for comparisons between groups (for comparisons between more than two groups). The threshold for statistical significance was set at threshold of $p<0.05$ and statistically significant differences are noted with an asterisk. P-values are otherwise noted with the value or labeled as not significant ("ns"). Error bars represent the mean value for the group +/- SD. Source Data are provided as a Source Data File. b, Table of Patients with clinical diagnoses and Patient group allocation based on results of clinical and ViroCap findings for single cell gene expression analyses. *H. flu* = *Haemophilus influenzae*; HRV = human rhinovirus; RSV = respiratory syncytial virus; *M. cat* = *Moraxella catarrhalis*; ADV = adenovirus; empiric rx = empiric antibacterial treatment; *GBS* = *Group B Streptococcus*; *S. pne* = *Streptococcus pneumoniae*; IAV = influenza A virus; *S. aur* = *Staphylococcus aureus*; cdh = congenital diaphragmatic hernia.

Supplementary Figure 2

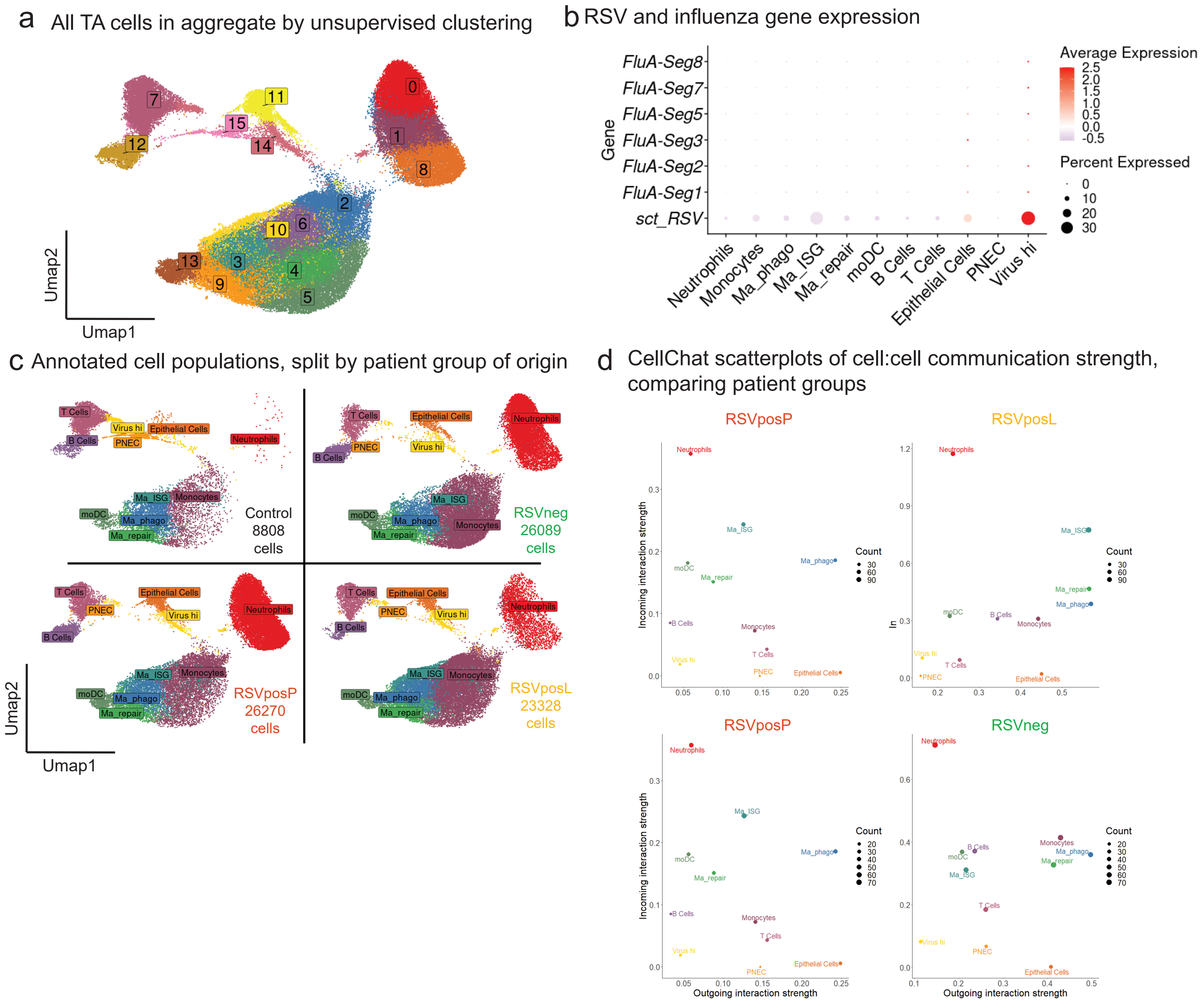

Supplementary Figure 2: Additional single cell analyses of aggregate data set. a, UMAP of all cells included in the final data set after initial unsupervised clustering analysis, labeled by cluster number. b, Dot plot of expression levels of RSV transcripts and IAV gene segments captured by single cell transcriptomics. Cell populations (x-axis) are labeled as in Figure 3a. The size of the dot indicates percent of cells within an individual cluster expressing a particular transcript, and the color indicates the average expression of the transcript within the cell population. c. UMAPs of all cells in the data set split by patient group and labeled by cell type as in Figure 3a. d, Scatterplot[24] comparing incoming and outgoing ligand-receptor communication strength amongst cell types (labeled as in Figure 3a) between Patient groups.

Supplementary Figure 3

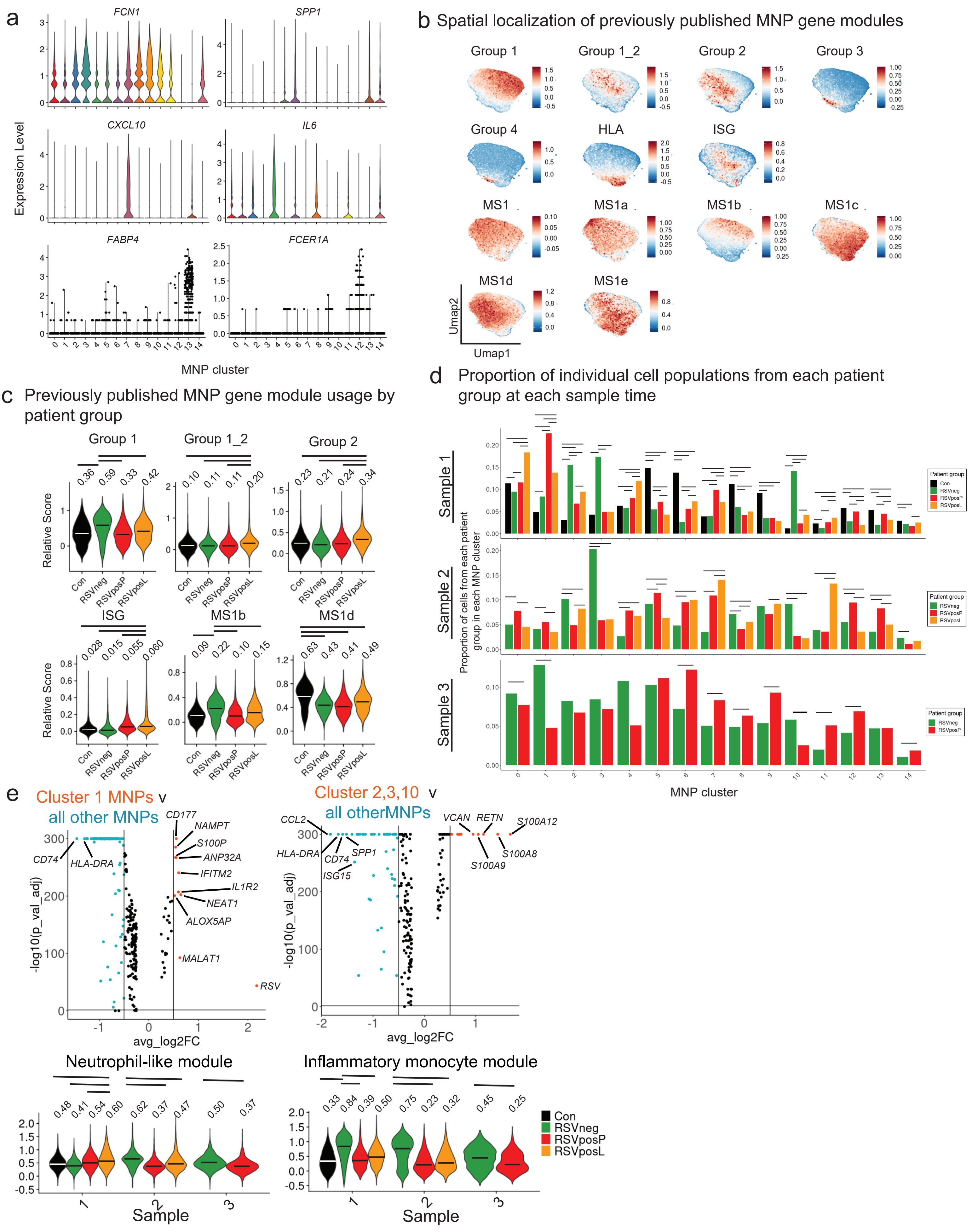

Supplementary Figure 3: Additional analyses of subclustered MNP populations. a,.Violin plots grouped by MNP cell cluster (labeled as in Figure 5a) of select MNP transcripts. b, Feature plots of module usage of previously published[30,32,33] gene sets in subclustered MNPs. c, Violin plots grouped by patient group of module usage of previously published[30,32,33] gene sets. Testing for statistical differences between groups was performed using a pairwise two-sided Wilcoxon rank sum test with continuity correction and p-value adjustment using the Benjamini & Hochberg correction. Significant differences (threshold p_{adj} <0.05) between groups are labeled with a bar (Group 1: C_v_RSVneg p_adj<2e-16, C_v_RSVposP p_adj=1.6e-6, C_v_RSVposL p_adj<2e-16, RSVposP_v_RSVneg p_adj<2e-16, RSVposL_v_RSVneg p_adj<2e-16, RSVposL_v_RSVposP p_adj<2e-16; Group1_2: C_v_RSVneg p_adj=0.03, C_v_RSVposP p_adj=1.1e-6, C_v_RSVposL p_adj<2e-16, RSVposP_v_RSVneg p_adj<2e-16, RSVposL_v_RSVneg p_adj<2e-16, RSVposL_v_RSVposP p_adj<2e-16; Group 2: C_v_RSVneg p_adj=1.1e-12, C_v_RSVposP p_adj=0.64, C_v_RSVposL p_adj<2e-16, RSVposP_v_RSVneg p_adj<2e-16, RSVposL_v_RSVneg p_adj<2e-16, RSVposL_v_RSVposP p_adj<2e-16; ISG: C_v_RSVneg p_adj<2e-16, C_v_RSVposP p_adj<2e-16, C_v_RSVposL p_adj<2e-16, RSVposP_v_RSVneg p_adj<2e-16, RSVposL_v_RSVneg p_adj<2e-16, RSVposL_v_RSVposP p_adj<2e-16; MS1b: C_v_RSVneg p_adj<2e-16, C_v_RSVposP p_adj=1.9e-7, C_v_RSVposL p_adj<2e-16, RSVposP_v_RSVneg p_adj<2e-16, RSVposL_v_RSVneg p_adj<2e-16, RSVposL_v_RSVposP p_adj<2e-16; MS1d: C_v_RSVneg p_adj<2e-16, C_v_RSVposP p_adj<2e-16, C_v_RSVposL p_adj<2e-16, RSVposP_v_RSVneg p_adj<2e-16, RSVposL_v_RSVneg p_adj<2e-16, RSVposL_v_RSVposP p_adj<2e-16). Median module usage within each patient group is denoted with a bar in the violin plot and further annotated numerically above the violin. Source Data are provided as a Source Data File. d. Bar plots of proportional abundance of patient group MNPs within individual MNP cell clusters (labeled as in Figure 5a) at each Sample time point. Testing for significant differences between

groups was performed using pairwise proportion test with continuity correction and p-value adjustment using Bonferroni correction (more than two groups compared) and a proportion test (two groups compared). Significant differences (threshold p_{adj} <0.05) between groups are labeled with a bar. Source Data are provided as a Source Data File. e, Volcano plot demonstrating DEGs between MNP cluster 1 (top, left) and MNP clusters 2, 3, and 10 (top, right) and all other MNPs. DGE testing was performed using Seurat's FindAllMarkers in default settings (Wilcoxon rank sum test with Bonferroni p-value adjustment) comparing cluster 1 and clusters 2,3, and 10 to all other MNPs. Violin plots (bottom) grouped by Sample time point and split by patient group of cluster 1 (Neutrophil-like; bottom left) and cluster 2, 3, and 10 (Inflammatory monocyte; bottom right) module usage. Testing for differences between groups was performed using a Kruskall-Wallis test as well as pairwise Wilcoxon rank sum test with p-value adjustment using the Benjamini & Hochberg correction (for comparisons amongst more than two groups) and an unpaired two-sided t test (for comparisons between two groups). Significant differences (threshold p_{adj} <0.05) between groups are labeled with a bar (All p_adj values are < 2e-16 for Neutrophil like Module comparisons, and with the exception of Sample 1 C_v_RSVposP, which had a p_adj value = 3e-14, all p_adj values are <2e-16 for Inflammatory Monocyte Module comparisons). Median module usage is indicated with a bar in the violin plot and numerically annotated above the violin. Source Data are provided as a Source Data File.

Supplementary Figure 4

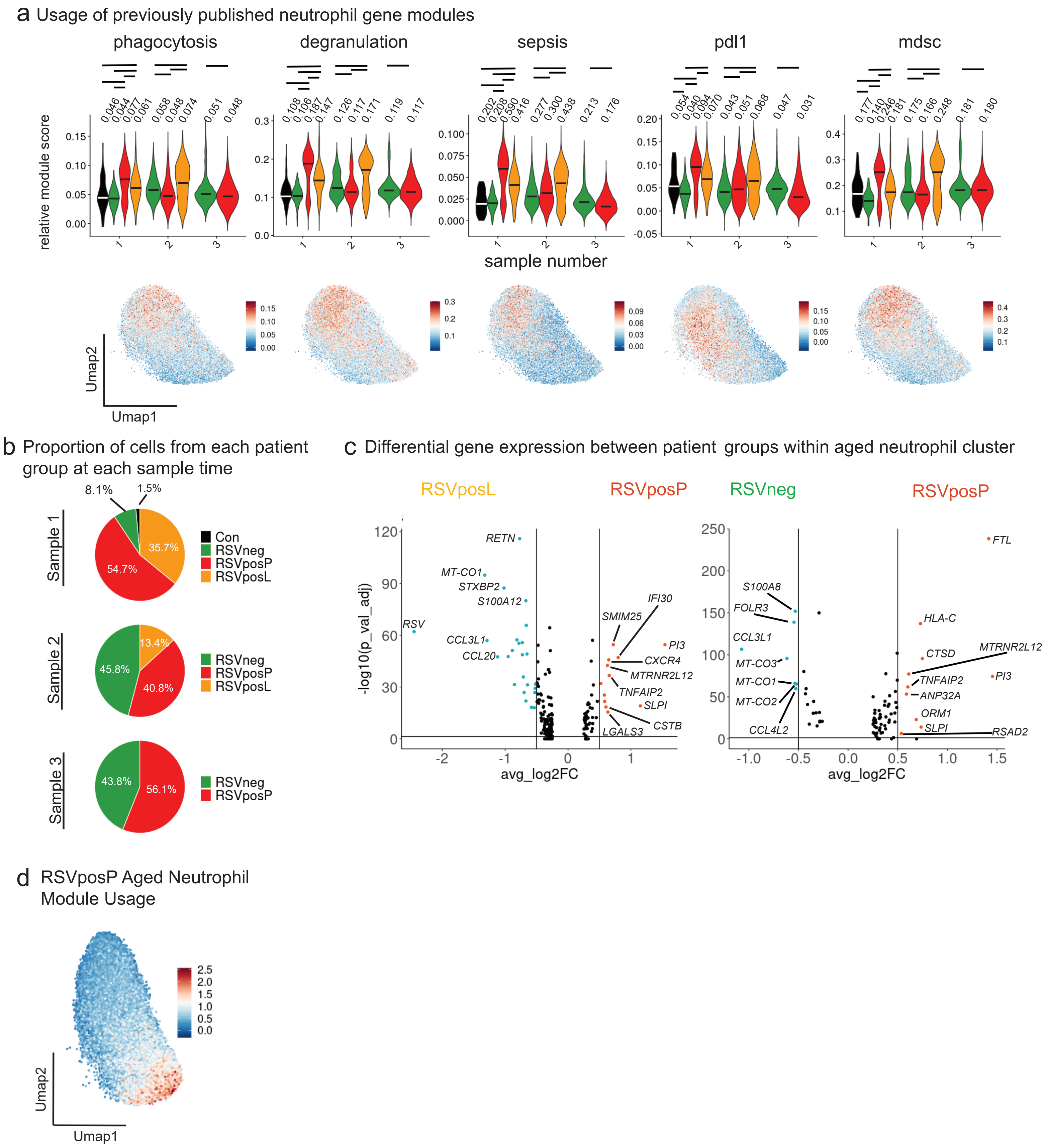

Supplementary Figure 4: Additional analyses of subsetted neutrophil populations. a, (Top) Violin plots of module usage of previously published neutrophil gene sets[29] . Testing for differences between groups was performed using a Kruskall-Wallis test as well as pairwise two-sided Wilcoxon rank sum test with p-value adjustment using the Benjamini & Hochberg correction (for comparisons amongst more than two groups) and an unpaired two-sided t test (for comparisons between two groups). Significant differences (threshold $p_{adj} <0.05$) between groups are labeled with a bar (Phagocytosis Sample 1: C_v_RSVneg p_adj=0.212, C_v_RSVposP p_adj=1.2e-6, C_v_RSVposL p_adj=0.0013, RSVposP_v_RSVneg p_adj<2e-16, RSVposL_v_RSVneg p_adj<2e-16, RSVposL_v_RSVposP p_adj<2e-16; Phagocytosis Sample 2: RSVneg_v_RSVposP p_adj<2e-16, RSVneg_v_RSVposL p_adj<2e-16, RSVposP_v_RSVposL p_adj<2e-16; Phagocytosis Sample 3: RSVneg_v_RSVposP p_adj<2e-16; Degranulation Sample 1: C_v_RSVneg p_adj=0.26, C_v_RSVposP p_adj=1.5e-10, C_v_RSVposL p_adj=1.3e-8, RSVposP_v_RSVneg p_adj<2e-16, RSVposL_v_RSVneg p_adj<2e-16, RSVposL_v_RSVposP p_adj<2e-16; Degranulation Sample 2: RSVneg_v_RSVposP p_adj<2e-16, RSVneg_v_RSVposL p_adj<2e-16, RSVposP_v_RSVposL p_adj<2e-16; Degranulation Sample 3: RSVneg_v_RSVposP p_adj=6e-16; Sepsis Sample 1: C_v_RSVneg p_adj=0.98, C_v_RSVposP p_adj=5.5e-16, C_v_RSVposL p_adj=1.9e-12, RSVposP_v_RSVneg p_adj<2e-16, RSVposL_v_RSVneg p_adj<2e-16, RSVposL_v_RSVposP p_adj<2e-16; Sepsis Sample 2: RSVneg_v_RSVposP p_adj=7.5e-8, RSVneg_v_RSVposL p_adj<2e-16, RSVposP_v_RSVposL p_adj<2e-16; Sepsis Sample 3: RSVneg_v_RSVposP p_adj<2e-16; PDL1 Sample 1: C_v_RSVneg p_adj=7.1e-5, C_v_RSVposP p_adj=2.2e-5, C_v_RSVposL p_adj=0.08, RSVposP_v_RSVneg p_adj<2e-16, RSVposL_v_RSVneg p_adj<2e-16, RSVposL_v_RSVposP p_adj<2e-16; PDL1 Sample 2: RSVneg_v_RSVposP p_adj<2e-16, RSVneg_v_RSVposL p_adj<2e-16, RSVposP_v_RSVposL p_adj<2e-16; PDL1 Sample 3: RSVneg_v_RSVposP p_adj<2e-16; MDSC Sample 1: C_v_RSVneg p_adj=0.00014,

C_v_RSVposP p_adj=6.5e-6, C_v_RSVposL p_adj=0.237, RSVposP_v_RSVneg p_adj<2e-16, RSVposL_v_RSVneg p_adj<2e-16, RSVposL_v_RSVposP p_adj<2e-16; Phagocytosis Sample 2: RSVneg_v_RSVposP p_adj<2e-16, RSVneg_v_RSVposL p_adj<2e-16, RSVposP_v_RSVposL p_adj<2e-16; Phagocytosis Sample 3: RSVneg_v_RSVposP p_adj=1.1e-5). Median module usage for each group is indicated with a bar in the violin plot and is annotated numerically above each violin. Source Data are provided as a Source Data File. (Bottom) Feature plots of module usage of previously published neutrophil gene sets[29]. b, Pie charts of proportion of patient group cells within the subsetted neutrophil population at each Sample time point. Source Data are provided as a Source Data File. c, Volcano plots demonstrating results of DGE testing between patient groups (RSVposP_v_RSVposL on left and RSVposP_v_RSVneg on right) within the aged neutrophil transcriptional phenotype. DGE testing was performed using Seurat's FindAllMarkers in default settings (two-sided Wilcoxon rank sum test with Bonferroni p-value adjustment). In each case a positive avg_log2FC indicates increased expression of transcripts within RSVposP aged neutrophils relative to the comparison Patient group aged neutrophils. Source Data are provided as a Source Data File. d, Feature plot of RSVposP aged neutrophil module usage (UMAP as in Figure 6a).

Supplementary Figure 5

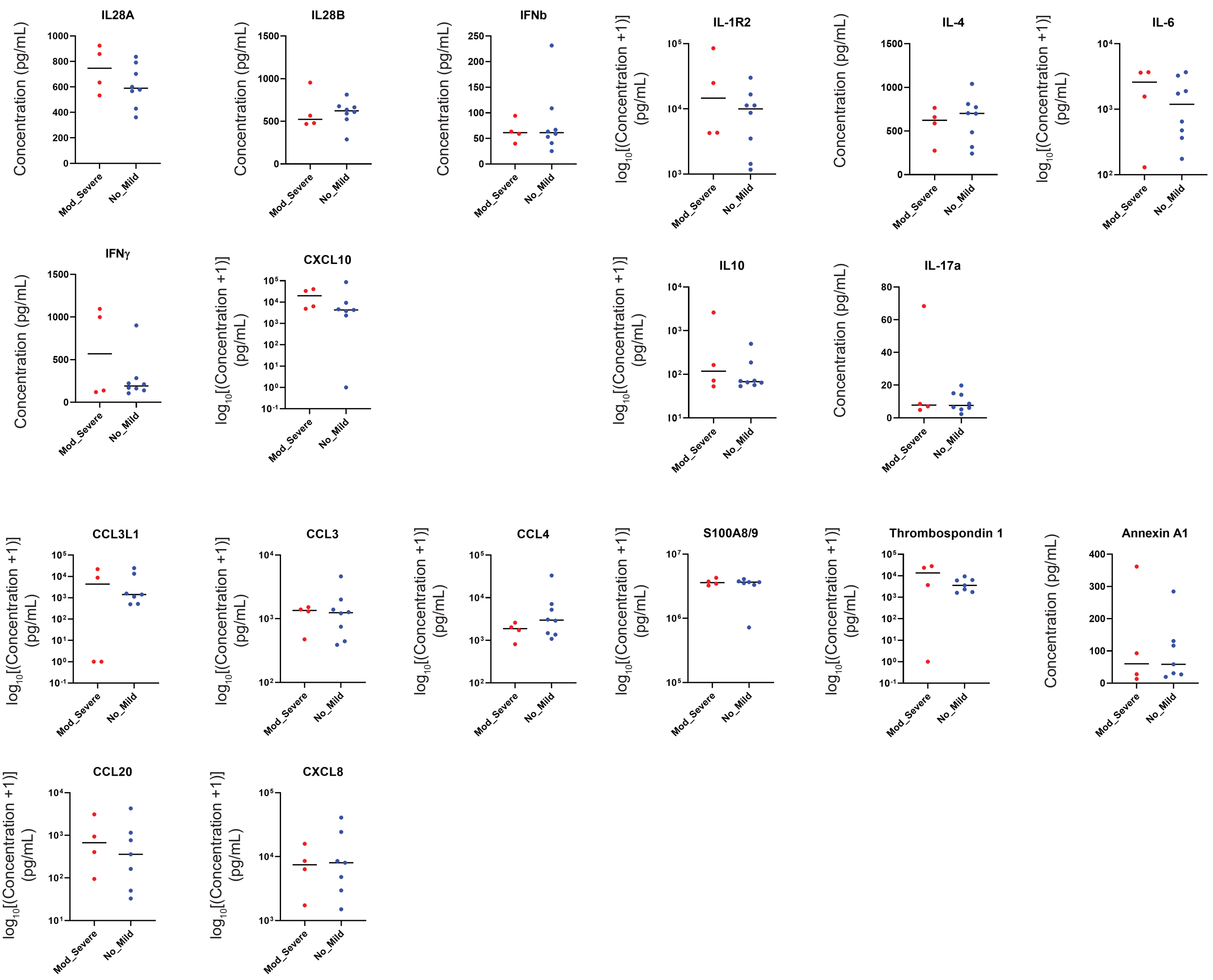

Supplementary Figure 5: Concentrations of additional analytes measured in tracheal aspirate fluid. Plots of additional analytes measured in tracheal aspirate samples from Patients enrolled in a different study at another center. The bar in the plot represents the median concentration of the analyte in each Patient group (n=4 Mod_Severe patients, n=7 patients for No_Mild analytes measured by ELISA and n=8 patients for No_Mild analytes measured by Luminex). Source Data are provided as a Source Data File.
