# Supplementary material for: Single cell transcriptomics identifies distinct profiles in pediatric acute respiratory distress syndrome
Source: Nat Commun. 2023 Jun 30;14:3870. doi: 10.1038/s41467-023-39593-0 (PMC10313703; doi:10.1038/s41467-023-39593-0)
Supplement: Supplementary file 3 — Description of Additional Supplementary Files [file 41467_2023_39593_MOESM3_ESM.pdf]

Description of Additional Supplementary Files

Supplementary Data 1: Detailed clinical variables extracted from the electronic medical record. OSI = Oxygen Saturation Index; MV = mechanical ventilation; ICU = Intensive Care Unit; PIP = Peak Inspiratory Pressure (in cmH_20); MAP = Mean Airway Pressure (in cmH_20); PEEP = Positive End Expiratory Pressure (in cmH_20); *H. flu* = *Haemophilus influenzae*; *M. cat* = *Moraxella catarrhalis*; *S. aureus* = *Staphylococcus aureus*; *K. pneumo* = *Klebsiella pneumoniae*; *E. coli* = *Escherichia coli*; *S. pneumo* = *Streptococcus pneumoniae*.

Supplementary Data 2: Gene Lists Used to Generate Module Scores.

Supplementary Data 3: Clinical variables for patients included from study performed at another center. RSV = Respiratory syncytial virus; ADV = Adenovirus; HMPV = Human metapneumovirus; HRV = Human rhinovirus; HCoV = Human coronavirus; H. flu = *Haemophilus influenzae*; MSSA = *Methicillin-susceptible Staphylococcus aureus*; GPC = Gram positive cocci; GNcb = Gram negative coccobacilli; MRSA = *Methicillin-resistant Staphylococcus aureus*.

Supplementary Data 4: Quality control metrics from individual single cell gene expression libraries included in aggregate dataset.
