# Supplementary material for: Single cell transcriptomics identifies distinct profiles in pediatric acute respiratory distress syndrome
Source: Nat Commun. 2023 Jun 30;14:3870. doi: 10.1038/s41467-023-39593-0 (PMC10313703; doi:10.1038/s41467-023-39593-0)
Supplement: Supplementary file 8 — Reporting Summary [file 41467_2023_39593_MOESM8_ESM.pdf]

nature portfolio

Corresponding author(s): Paul G. Thomas
Tim Flerlage

Last updated by author(s): 05.17.2023

Reporting Summary

Nature Portfolio wishes to improve the reproducibility of the work that we publish. This form provides structure for consistency and transparency in reporting. For further information on Nature Portfolio policies, see our Editorial Policies and the Editorial Policy Checklist.

Statistics

For all statistical analyses, confirm that the following items are present in the figure legend, table legend, main text, or Methods section.

n/a	Confirmed	
☐	☒	The exact sample size (*n*) for each experimental group/condition, given as a discrete number and unit of measurement
☐	☒	A statement on whether measurements were taken from distinct samples or whether the same sample was measured repeatedly
☐	☒	The statistical test(s) used AND whether they are one- or two-sided<br>*Only common tests should be described solely by name; describe more complex techniques in the Methods section.*
☐	☒	A description of all covariates tested
☐	☒	A description of any assumptions or corrections, such as tests of normality and adjustment for multiple comparisons
☐	☒	A full description of the statistical parameters including central tendency (e.g. means) or other basic estimates (e.g. regression coefficient) AND variation (e.g. standard deviation) or associated estimates of uncertainty (e.g. confidence intervals)
☐	☒	For null hypothesis testing, the test statistic (e.g. *F*, *t*, *r*) with confidence intervals, effect sizes, degrees of freedom and *P* value noted<br>*Give P values as exact values whenever suitable.*
☒	☐	For Bayesian analysis, information on the choice of priors and Markov chain Monte Carlo settings
☒	☐	For hierarchical and complex designs, identification of the appropriate level for tests and full reporting of outcomes
☒	☐	Estimates of effect sizes (e.g. Cohen's *d*, Pearson's *r*), indicating how they were calculated

Our web collection on statistics for biologists contains articles on many of the points above.

Software and code

Policy information about availability of computer code

Data collection	Data were collected and stored using Microsoft Excel
Data analysis	R v4.0.2 and 4.1.2<br>Seurat v4.0.3<br>sctransform v0.3.2<br>ggplot2 v3.3.5 and v3.4.0<br>dplyr v1.0.7 and v1.1.0<br>patchwork v1.1.1 and v 1.1.2<br>CellChat v1.1.3<br>ggrepel v0.9.1 and v 0.9.3<br>Kraken2 v2.0.7b<br>Bracken v2.5<br>Pavian v0.8.4<br>pROC v.1.18.0<br>VarScan v2.3.9<br>GraphPad 8.4.3<br>SOFTMAX Pro Software 7.1.4

For manuscripts utilizing custom algorithms or software that are central to the research but not yet described in published literature, software must be made available to editors and reviewers. We strongly encourage code deposition in a community repository (e.g. GitHub). See the Nature Portfolio guidelines for submitting code & software for further information.

Data

Policy information about availability of data

All manuscripts must include a data availability statement. This statement should provide the following information, where applicable:

- Accession codes, unique identifiers, or web links for publicly available datasets
- A description of any restrictions on data availability
- For clinical datasets or third party data, please ensure that the statement adheres to our policy

The raw sequencing data generated in this study have been deposited in the Sequence Read Archive database under accession code PRJNA971535. The single cell analysis data generated in this study used to generate figures are provided in the Source Data file.

Research involving human participants, their data, or biological material

Policy information about studies with human participants or human data. See also policy information about sex, gender (identity/presentation), and sexual orientation and race, ethnicity and racism.

Reporting on sex and gender	The term gender is not used. The following statement appears in our methods section: "Patient sex was not considered in the study design; however, these data were collected as listed in the EMR for the patient after obtaining informed consent for participation." These data appear in disaggregated form in Supplementary Table 1, which lists all demographic and clinical data for the patients enrolled on the study. We have obtained approval from the IRB of record to publish these data in disaggregated form. We additionally include the following statement in our discussion section as this was a single-centered study: "Finally, there were differences in demographic variables, including age, sex, and race, between patient cohorts, which may contribute to differences in immune responses between individuals. Thus, our findings, which were developed using analyses of samples obtained from patients admitted to a single center, will require future validation in a larger cohort in which these demographic variables can be better balanced between comparator groups."
Reporting on race, ethnicity, or other socially relevant groupings	We collected data on race and reported it in Supplementary Table 1. These data were not used as proxies for anything. We have obtained approval from the IRB of record to publish these data in disaggregated form. We additionally include the following statement in our discussion section as this was a single-centered study: "Finally, there were differences in demographic variables, including age, sex, and race, between patient cohorts, which may contribute to differences in immune responses between individuals. Thus, our findings, which were developed using analyses of samples obtained from patients admitted to a single center, will require future validation in a larger cohort in which these demographic variables can be better balanced between comparator groups."
Population characteristics	The analysis includes samples obtained from patients aged 0-2 years of age. As above, in our discussion section, we specifically note certain differences in key demographic data as potential limitations of the study: "Finally, there were differences in demographic variables, including age, sex, and race, between patient cohorts, which may contribute to differences in immune responses between individuals. Thus, our findings, which were developed using analyses of samples obtained from patients admitted to a single center, will require future validation in a larger cohort in which these demographic variables can be better balanced between comparator groups."
Recruitment	Patients were identified and recruited through daily review of the pediatric intensive care unit (PICU) census. Patients newly admitted to the PICU who required endotracheal intubation and mechanical ventilation were screened for study eligibility. If a patient met study criteria, their legal guardians were approached for informed consent for study participation. Over the study period, the PICU census was screened daily and if a patient met criteria, then they were approached. We had one refusal to participate, thus the potential for self-selection bias is minimal.
Ethics oversight	University of Tennessee Institutional Review Board (IRB) (IRB Number 17-504-05744-XP STJUDE)<br>St. Jude Children's research Hospital IRB (IRB Number 505 Pro00008351)

Note that full information on the approval of the study protocol must also be provided in the manuscript.

Field-specific reporting

Please select the one below that is the best fit for your research. If you are not sure, read the appropriate sections before making your selection.

[x] Life sciences [ ] Behavioural & social sciences [ ] Ecological, evolutionary & environmental sciences

For a reference copy of the document with all sections, see nature.com/documents/nr-reporting-summary-flat.pdf

Life sciences study design

All studies must disclose on these points even when the disclosure is negative.

Sample size	There were no sample size calculations performed prior to the study as appropriate methodologies for sample size calculations for multi-sample single cell RNA sequencing studies is still an evolving field. Prospective enrollment took place with subjects allocated according to clinical diagnosis and analysis took place comparing the single cell RNA-sequencing findings between clinical patient groups.
Data exclusions	No cells were able to be isolated from one subject's first TA sample. As single cell RNA sequencing was the primary objective of this study, this

	subject's data was not included in the final analysis. This is reported in the manuscript.
Replication	As this was a prospective observational study using single cell RNA-sequencing on primary human samples, replication of reported results from individual samples was not possible and was not attempted. However, patients were enrolled and grouped into individual cohorts/ groups based on clinical severity and underlying etiology by study design, which enabled statistical comparisons between cohorts/groups that were composed of multiple patients with a similar clinical phenotype.
Randomization	As this was a prospective observational study without an intervention, there was no randomization in this study.
Blinding	As this was a prospective observational study without an intervention, there was no blinding in this study.

Reporting for specific materials, systems and methods

We require information from authors about some types of materials, experimental systems and methods used in many studies. Here, indicate whether each material, system or method listed is relevant to your study. If you are not sure if a list item applies to your research, read the appropriate section before selecting a response.

Materials & experimental systems

n/a	Involved in the study
☒	☐ Antibodies
☒	☐ Eukaryotic cell lines
☒	☐ Palaeontology and archaeology
☒	☐ Animals and other organisms
☐	☒ Clinical data
☒	☐ Dual use research of concern
☒	☐ Plants

Methods

n/a	Involved in the study
☒	☐ ChIP-seq
☒	☐ Flow cytometry
☒	☐ MRI-based neuroimaging

Clinical data

Policy information about clinical studies

All manuscripts should comply with the ICMJEguidelines for publication of clinical research and a completedCONSORT checklist must be included with all submissions.

Clinical trial registration	Not applicable as this was not a clinical trial.
Study protocol	A full study protocol is available and can be supplied with this manuscript.
Data collection	Data were collected from the electronic medical record at LeBonheur Children's Hospital following participant enrollment, which took place from March 2018 to February 2020.
Outcomes	Because this was a observational study, there were no primary or secondary outcomes.
